# Supplementary material for: Combined Approach of GWAS and Phylogenetic Analyses to Identify New Candidate Genes That Participate in Arabidopsis thaliana Primary Root Development Using Cellular Measurements and Primary Root Length
Source: Plants (Basel). 2022 Nov 18;11(22):3162. doi: 10.3390/plants11223162 (PMC9697774; doi:10.3390/plants11223162)
Supplement: Supplementary file 1 [file plants-11-03162-s001.zip › Figure supplementary S1-S6.pdf]

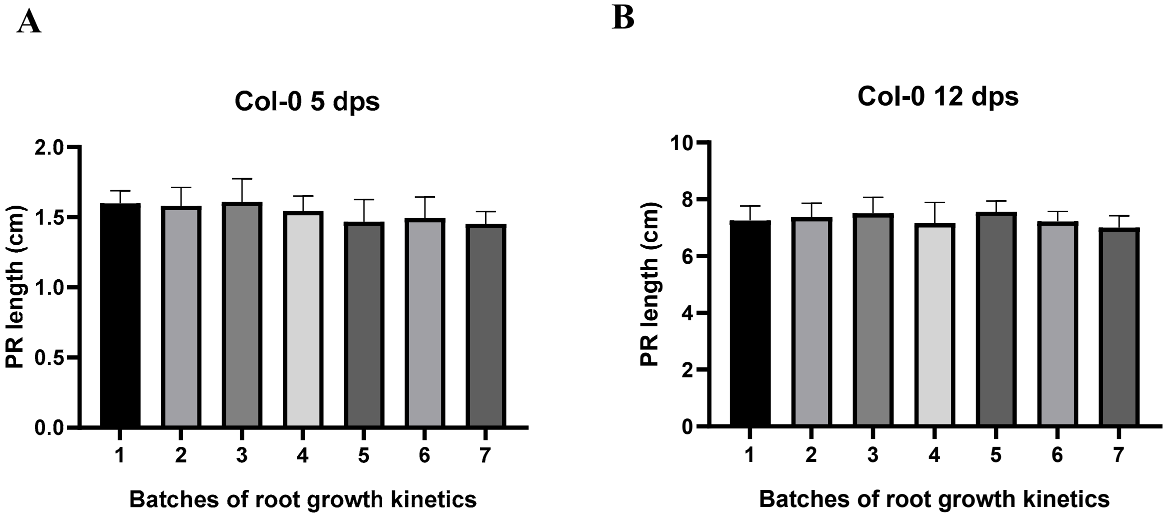

Figure S1. Primary root length of Col-0 at 5 (A) and 12 dps (B) used as internal control in the seven batches of the time course growth of the 124 accessions. No statistical differences (*one-way ANOVA* $P < 0.05$; HSD-test, $P < 0.05$) were observed between batches.

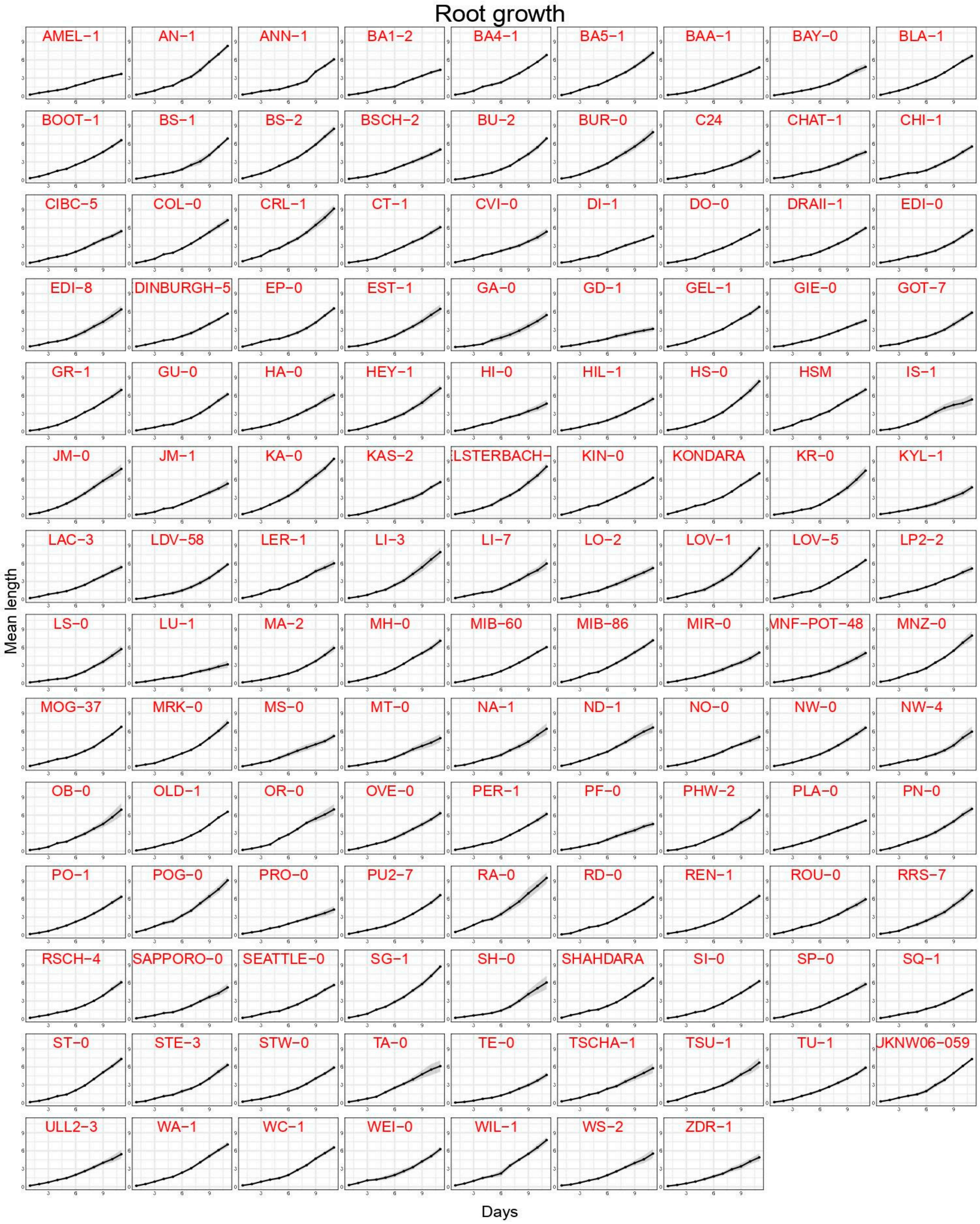

Figure S2. The primary root time course growth of the 124 accessions performed during 12 dps.

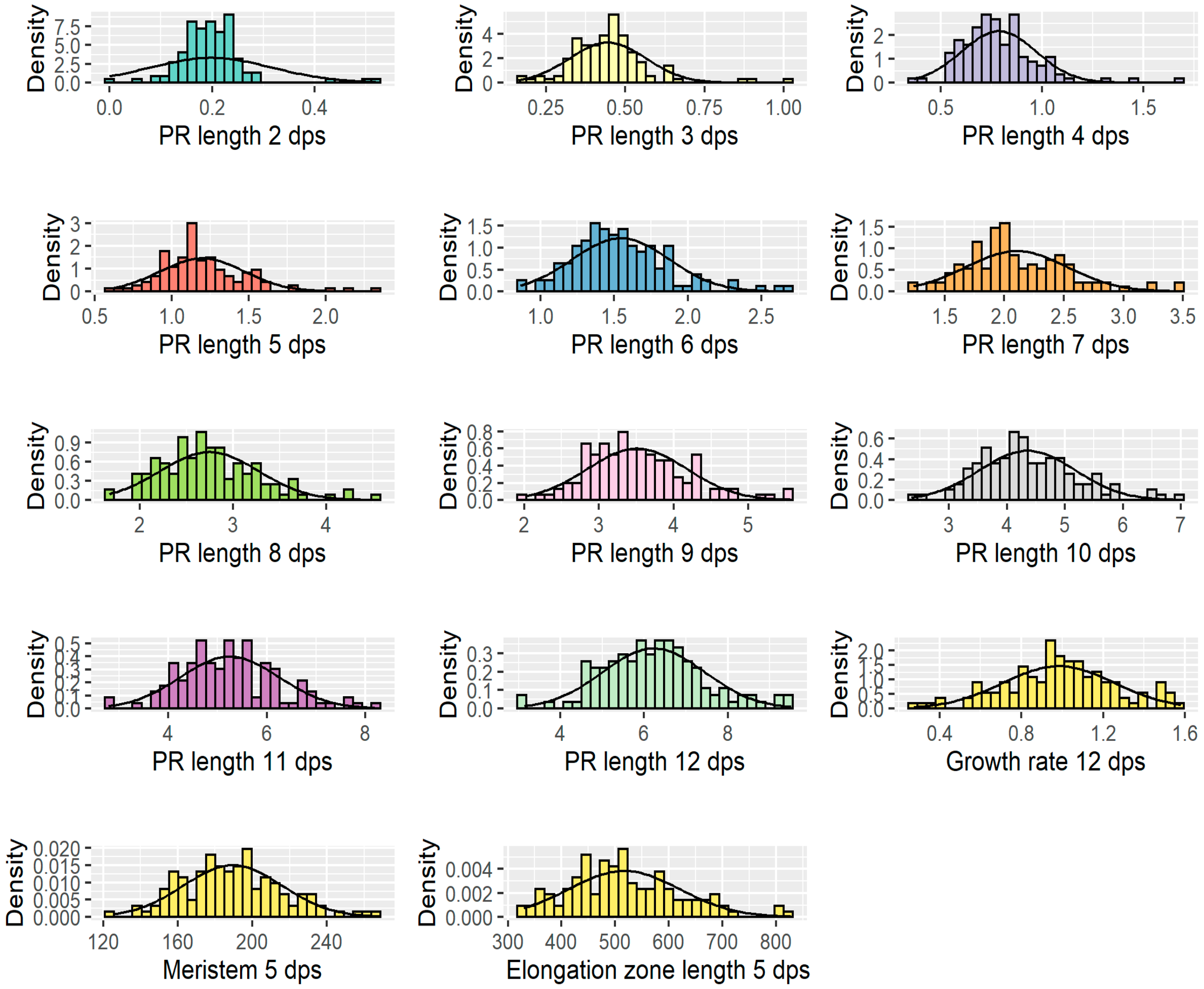

Figure S3. Histograms representing the density distribution of each trait analysed.

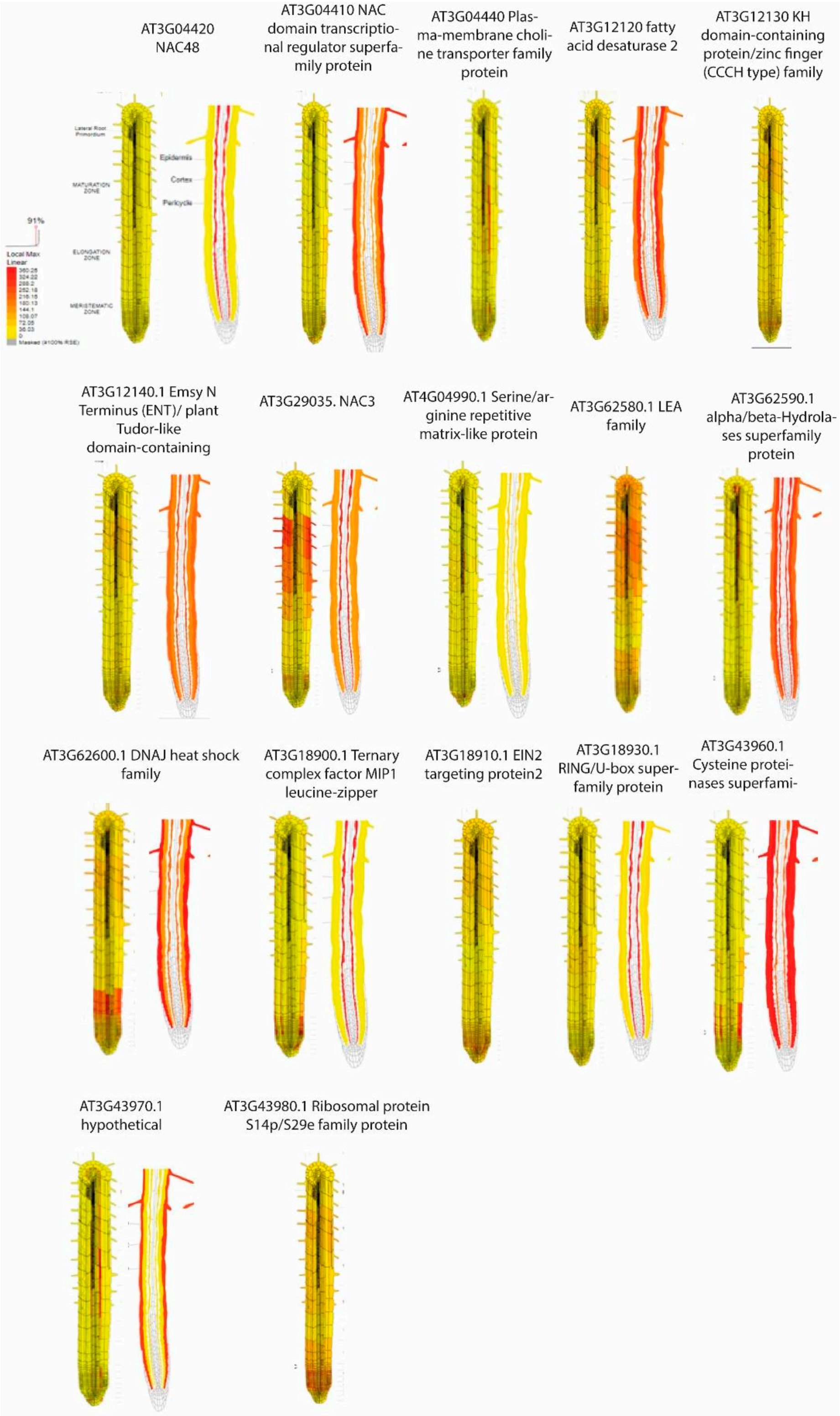

Figure S4. Root expression of genes found in GWAS. The images were retrieved from the Plant and Tissue and Experiment eFP Viewer (http://bar.utoronto.ca/eplant/)

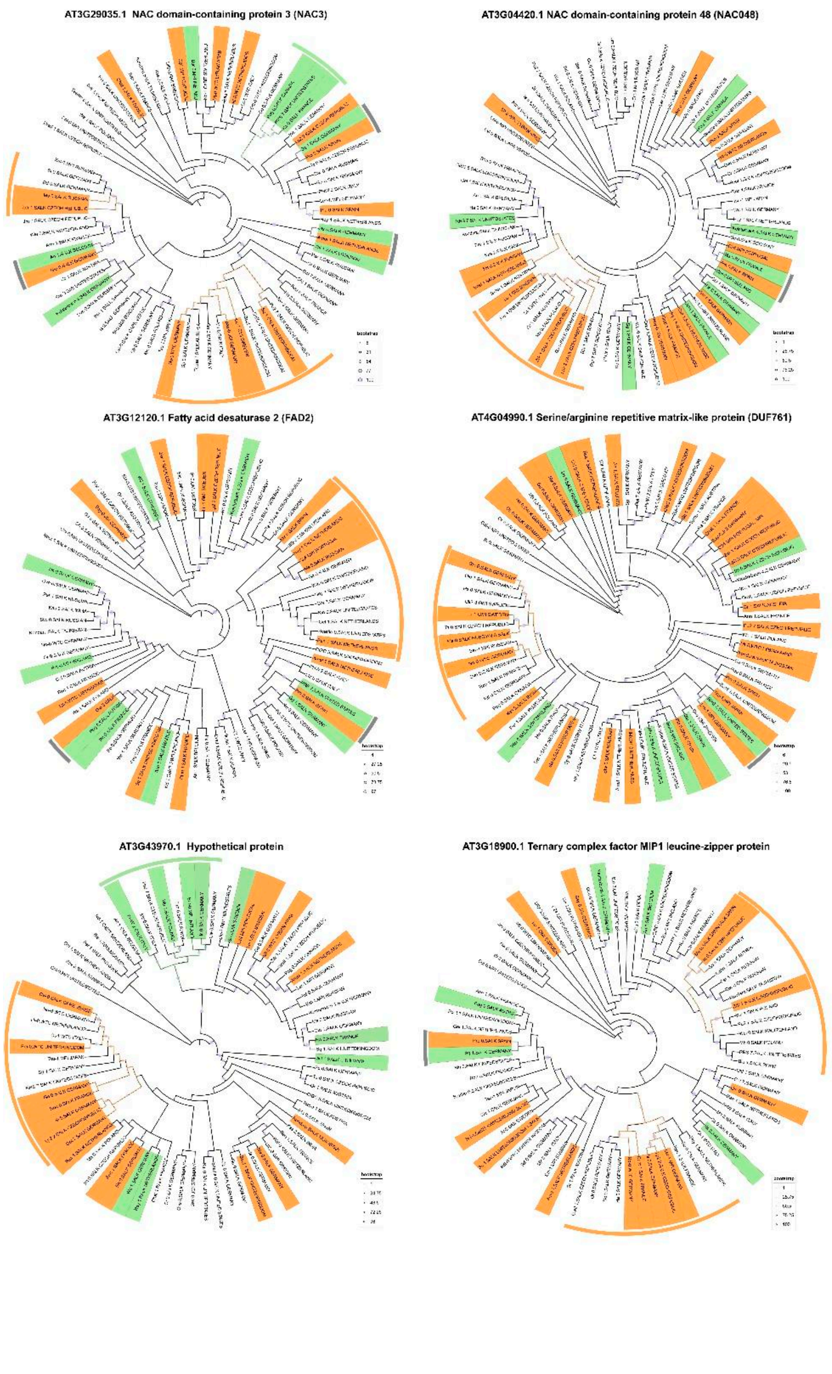

Figure S5. Phylogenetic trees of the genes with the highest score found in GWAS and based on the four traits analysed

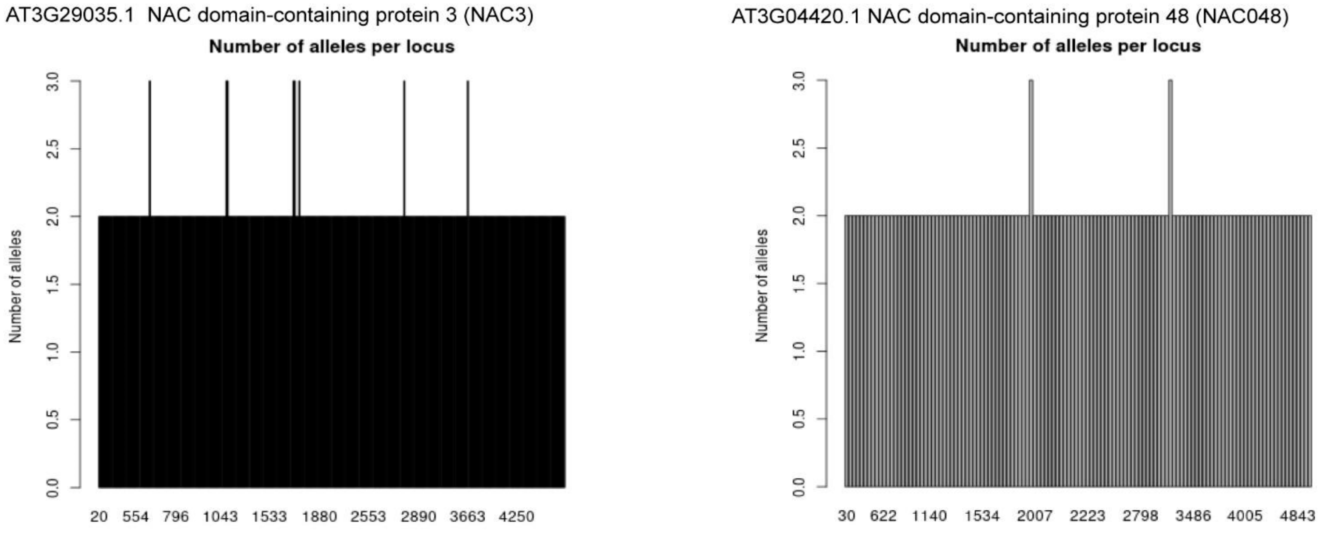

Figure S6. Number of alleles per locus derived from the alignment of homologs for NAC3 and NAC048 from 79 accessions. (A) NAC3 has 92 SNPs yielding 2 alleles and 3 SNPs related to 3 additional alleles in exon 3 (701 and 1470pb). (B) NAC048 has 3 SNPs with 2 alleles in exon 3 (578 and 718pb) and 2 SNPs with 2 other alleles in exon 5 (between 1080 and 1157pb). The analysis was made using a adegenet R package.
