# Supplementary material for: Combined Approach of GWAS and Phylogenetic Analyses to Identify New Candidate Genes That Participate in Arabidopsis thaliana Primary Root Development Using Cellular Measurements and Primary Root Length
Source: Plants (Basel). 2022 Nov 18;11(22):3162. doi: 10.3390/plants11223162 (PMC9697774; doi:10.3390/plants11223162)
Supplement: Supplementary file 1 [file plants-11-03162-s001.zip › Table S4. Root expression.pdf]

Table S6. *Root expression of genes found in GWAS. ND= Not determined*

**Gene**		**Root expression**	**Mutant phenotype in root**	**Reference**
AT3G04420.1	NAC048	Cambium of vascular tissues Late-stage differentiating root hair epidermal cells	Overexpression leads to vascular disorganization	Yang et al., 2019 ePLANT
AT3G04410.1	NAC domain transcriptional regulator superfamily protein	Cortex, epidermis pericycle, lateral root cap	ND	ePLANT
AT3G04430.1	NAC domain-containing protein 49	ND	ND	ePLANT
AT3G04440.1	Plasma-membrane choline transporter family protein	Endodermis in maturation zone	ND	ePLANT
AT3G04443.1	hypothetical protein	PR 1 day seedling	ND	EFP BROWSER
AT3G12120.1	fatty acid desaturase 2	Cortex, epidermis pericycle, meristem, differentiation zone	*fad2* loss of function has short PR length that WT	Zhang et al., 2012, ePLANT
AT3G12130.1	KH domain-containing protein/zinc finger (CCCH type) family protein	Cortex, epidermis pericycle, meristem, differentiation zone	ND	Yan et al., 2017, ePLANT
AT3G12140.1	Emsy N Terminus (ENT)/ plant Tudor-like domain-containing protein	Cortex, epidermis.	ND	ePLANT
AT3G29034.1	Transmembrane protein	PR 1-day seedling	ND	EFP BROWSER
AT3G29035.1	NAC domain-containing protein 3	Root tip, vasculature, maturation zone	ND	Balazadeh et al., 2011, ePLANT
AT4G04990.1	Serine/arginine repetitive matrix-like protein (DUF761)	Vasculature, procambium, pericycle	ND	ePLANT
AT3G62570.1	Tetratricopeptide repeat (TPR)-like superfamily protein	PR 1-day seedling	ND	EFP BROWSER

AT3G62580.1	Late embryogenesis abundant protein (LEA) family protein	Vasculature, Maturation zone, cortex, epidermis pericycle, meristem, Elongation zone	ND	ePLANT
AT3G62590.1	PLIP3 alpha/beta-Hydrolases superfamily protein	Vasculature, cortex, epidermis pericycle,	ND	ePLANT
AT3G62600.1	DNAJ heat shock family protein, ERdj3B	Elongation zone, cortex, epidermis pericycle, Quiescent center	ND	ePLANT
AT3G18900.1	Ternary complex factor MIP1 leucine-zipper protein	Meristem, pericycle	ND	ePLANT
AT3G18905.1	F-box/associated interaction domain protein	ND	ND	EFP BROWSER
AT3G18910.1	EIN2 targeting protein2	Quiescent centre, Differentiating lateral root cap cells	The overexpression led to shorter PR in the presence of ethylene	Qiao et al., 2008
AT3G18930.1	RING/U-box superfamily protein	Stele	ND	ePLANT
AT3G43960.1	Cysteine proteinases superfamily protein	Expression between meristem and elongation zone. Early-stage differentiating root hair epidermal cells, cortex, epidermis pericycle,	Mutant exhibits shorter root hairs under phosphate-deficient conditions.	Lin et al., 2011
AT3G43970.1	hypothetical protein	Vasculature, epidermis	ND	ePLANT
AT3G43980.1	Ribosomal protein S14p/S29e family protein	Vasculature, meristem	ND	ePLANT
